# Huge Intrathoracic Malignant Peripheral Nerve Sheath Tumor in an Adolescent with Neurofibromatosis Type 1

**DOI:** 10.1155/2014/951252

**Published:** 2014-05-25

**Authors:** Jong Hyung Yoon, Hyun-Sung Lee, Jong In Chun, Seog-Yun Park, Hyeon Jin Park, Byung-Kiu Park

**Affiliations:** ^1^Center for Pediatric Oncology, National Cancer Center, 323 Ilsan-ro, Ilsandong-gu, Goyang-si, Gyeonggi-do 410-769, Republic of Korea; ^2^Center for Lung Cancer, National Cancer Center, 323 Ilsan-ro, Ilsandong-gu, Goyang-si, Gyeonggi-do 410-769, Republic of Korea; ^3^Department of Pathology, National Cancer Center, 323 Ilsan-ro, Ilsandong-gu, Goyang-si, Gyeonggi-do 410-769, Republic of Korea

## Abstract

Malignant peripheral nerve sheath tumor (MPNST) is a rare soft tissue malignancy usually found in patients with neurofibromatosis type 1 (NF1) with a poor outcome. Although MPNST can be found in any part of the body including head and neck or extremities, intrathoracic MPNST with or without NF1 is uncommon, especially in children or adolescents. Reported herein is a case of huge intrathoracic MPNST in a 16-year-old girl with NF1, and a brief review of the literature.

## 1. Introduction

Malignant peripheral nerve sheath tumor (MPNST) is a rare soft tissue malignancy generally encountered during adulthood and only 10–20% is found in children and adolescents [1–4]. Many cases of this tumor arise in the patients with neurofibromatosis type 1 (NF1) with a poor outcome [1, 3, 4]. Although MPNST can be found in any part of the body including extremities, head and neck, trunk, or retroperitoneum [1, 2], intrathoracic MPNST with or without NF1 is uncommon, only with several reported adult cases [5–15]. In children or adolescents, only a few cases have been reported up to date [16–20].

Here, we report our experience in a rare case of huge intrathoracic MPNST in a 16-year-old adolescent with NF1. A review of the pertinent literature is included.

## 2. Case Presentation

**Figure 1:**
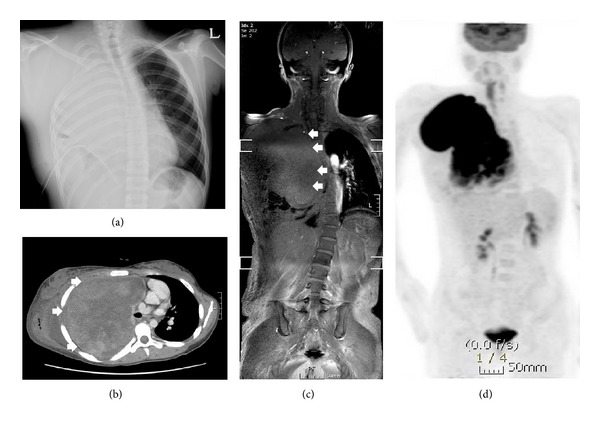
The patient's chest radiography shows a large thoracic mass with total collapse of right lung (a). Her chest CT scan (b) and spine MR imaging (c) also show a large intrathoracic hyperdense mass (white arrows) in the right chest, resulting in prominent scoliosis and cardiac deviation. Her ^18^F-FDG PET/CT scan showed highly increased FDG uptake (SUV_max⁡_ = 6.9) in this mass, suggesting highly malignant tumor (d).

A 16-year-old girl visited our hospital because of progressive chest discomfort and respiratory difficulties that started a month prior to the visit. Physical examination performed during her visit revealed multiple café-au-lait spots and cutaneous neurofibromas indicating NF1 in the patient as well as in the patient's mother. Her height and weight were 153 cm (5–10 percentile) and 48.4 kg (10–25 percentile), respectively. Her Tanner stage was 4. Her blood pressure, pulse rate, respiratory rate, and body temperature were 136/66 mmHg, 114/min, 24/min, and 36.5°C, respectively. Her chest radiography and chest computed tomography (CT) images revealed a large mass occupying the right thoracic cavity with multiple pleural nodules suggesting metastasis (Figures 1(a) and 1(b)). Scoliosis was associated with the tumor and multiple neurofibromas on magnetic resonance imaging of the whole spine (Figure 1(c)). The findings were suggestive of highly malignant tumor on positron emission tomography (PET)/CT imaging (Figure 1(d)) with a maximum standardized uptake value (SUV_max⁡_) of 6.9. Her* NF1* gene mutation analysis revealed no known overt mutation except silent mutation in C369G without change of amino acids.

**Figure 2:**
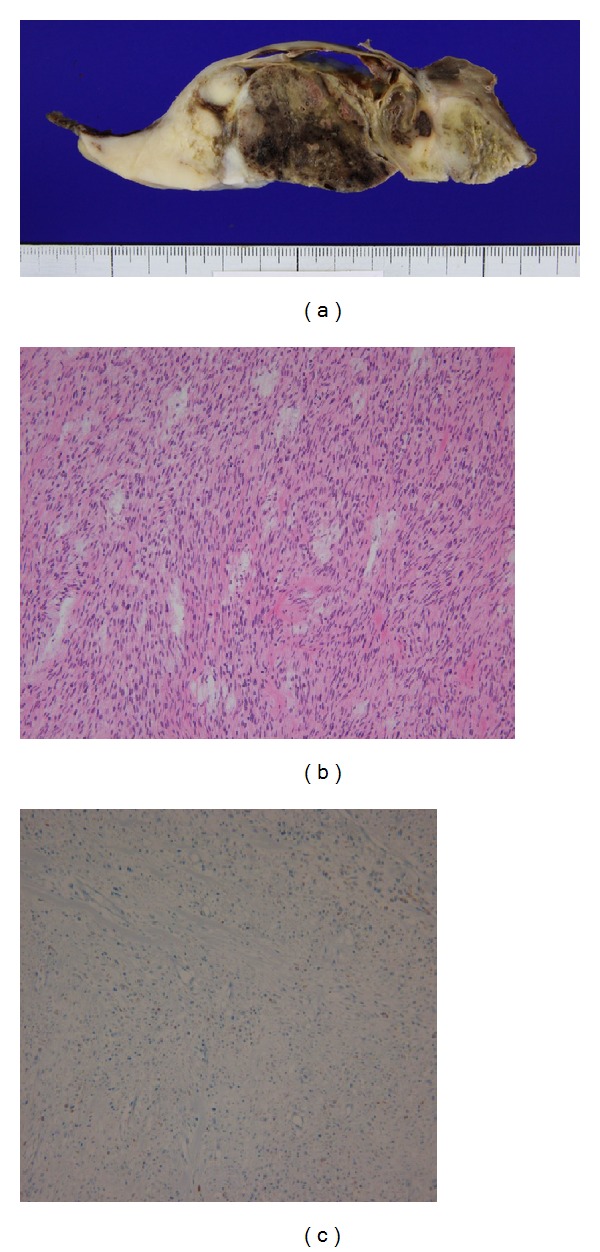
Macroscopic appearance of the cut surface of the resected tumor shows some hemorrhage and necrosis in the center. It was 22 × 17 × 9 cm in size (a). Microscopically, the tumor is composed of homogenous spindle cells (hematoxylin and eosin, ×100) (b). Immunohistochemical stains showing positivity to neuron-specific enolase (c), consistent with MPNST.

She underwent immediate surgery (grossly total resection of the tumor and chest wall reconstruction with patch graft) without diagnostic biopsy because total resection of the tumor seemed feasible. Intraoperatively, the tumor was encapsulated and showed adhesion to pleura with somewhat of effusion and incomplete fissure. Brachial plexus, vagus nerve, and phrenic nerve were functionally saved without tumor involvement. Macroscopically, the resected tumor was 22 × 17 × 9 cm in size (Figure 2(a)). Pulmonary metastasis was not detected in intraoperative findings. Microscopic features of the tumor showed many spindle-shaped cells with pale, eosinophilic cytoplasm (Figure 2(b)). Immunohistochemically, tumor cells expressed neuron-specific enolase (Figure 2(c)) and CD68 (focal) but not desmin, CD34, and smooth muscle actin, which were consistent with a diagnosis of MPNST.

After the surgery, she received 6,600 cGy of tomotherapy to the right whole lung field and pleura. Subsequently, she received 6 courses of chemotherapy consisting of vincristine, doxorubicin, and cyclophosphamide, alternating with ifosfamide and etoposide (VDC/IE) [21]. However, her residual pleural metastasis showed no definite response to adjuvant therapies and showed rapid progression with newly appearing pulmonary nodules. She developed a large amount of malignant pericardial effusion associated with enlarged pulmonary metastases after the sixth course of chemotherapy. Despite supportive measures including pericardiocentesis, she died of progressive disease at 9 months after the surgery.

## 3. Discussion

NF1, formerly called von Recklinghausen disease, is an autosomal dominant neurocutaneous disorder, with an estimated incidence of 1 in 3000 births [4, 16]. NF1 is commonly associated with higher incidence in many kinds of benign or malignant tumors, such as optic pathway glioma, chronic myeloid leukemia, or pheochromocytoma, neurofibroma, and MPNST [22], and is related to mutations in* NF1* gene located in chromosome 17q11.2. Because* NF1* gene product named neurofibromin acts as a negative regulator in Ras signal transduction pathway, mutation in* NF1* gene is related to tumor development and results in malignancies [22, 23].

MPNST is a rare soft tissue malignancy comprising about 5–10% of all soft tissue sarcomas [1–4]. Its incidence in healthy people is approximately 0.001%, but its incidence is much higher in NF1 patients (2~5%) [3, 4]. Inversely, MPNST is one of the most common malignancies in NF1 patients and 50% of MPNST arises in NF1 patients [1–3]. MPNST can be found in any part of the body, including extremities (40%), trunk (22%), head and neck (20%), and retroperitoneal visceral area (15%) [1, 2]. However, intrathoracic or mediastinal MPNST is very rare. Although 14 cases of intrathoracic MPNST with (8) or without (6) NF1 were reported previously in English-language literature, most of them were in adult patients with a median age of 40 (54.5 without and 39.5 with NF1) years [5–15] and only four cases with NF1 (including one with angiosarcoma component) and two without NF1 of children or adolescents have been reported (Table 1) [16–20]. We were unable to find differences between the patients with and without NF1 because of limited number of the patients. Despite its rarity, intrathoracic MPNSTs in patients with NF1 are thought to be originated from the plexiform neurofibromas (PNs) of thoracic nerves or vagus nerve [13]. Four (including one with angiosarcoma component) of them with NF1 had no metastasis at diagnosis, and only two survived longer than 12 months. Our patient had a tumor huge in size compared to those of previous reports with multiple pleural nodules suggesting metastasis at diagnosis, which indicates delayed diagnosis despite her NF1 stigmata. It is well known that MPNST associated with NF1 shows a poor response to chemotherapy or radiotherapy and the prognosis with residual tumor or metastasis is dismal [1, 2, 18]. Although doxorubicin/ifosfamide- (AI-) based chemotherapy is known to be somewhat effective for adult MPNST and some pediatric cases [4, 23, 24], there is no known standard chemotherapy regimen for treatment of MPNST. Since MPNST with NF1 usually shows poor response to conventional chemotherapy we used 5-drug chemotherapy to treat our patient because VDC/IE regimen for the Ewing sarcoma or rhabdomyosarcoma may show a better response to MPNST than AI-based regimen [4, 21, 23, 24]. However, our patient also showed no response of tumor to adjuvant therapies and died of disease progression.

Wide excision of the tumor is one of the most important prognostic factors in MPNST [2]. Because some of the large intrathoracic malignant tumors cannot be removed completely due to adjacent critical vital organs, early detection of intrathoracic MPNST is very important for long-term survival of the patients with NF1 [20]. Although some assessment guidelines for children with NF1 including annual clinical evaluation of spine were suggested [25], no specific evaluation strategy for surveillance of MPNST in the patients with NF1 is known [20, 22]. Because MPNST in NF1 patient is usually transformed from PNs and they have similar initial symptoms and signs, it is very important to distinguish small MPNST from PN for early detection of MPNST [20]. Recently, some reports suggest that ^18^FDG-PET/CT (with various cutoff SUV_max⁡_ of 2.5~3.5) can be a useful diagnostic tool for early detection and good prognosis of MPNST in NF1 patients [20, 26, 27]. However, considering no solid cutoff value of SUV_max⁡_ and risk of radiation exposure after ^18^FDG-PET/CT, further investigation for adequate schedule or strategy using PET/CT for early detection and rapid intervention of MPNST in NF1 should be warranted.

The authors report a rare case of huge intrathoracic MPNST in a 16-year-old girl with NF1. Considering its location and poor prognosis without complete resection, a screening strategy for early detection and intervention of MPNST in NF1 should be investigated.

## Figures and Tables

**Table 1:** Reported cases of children and adolescents with intrathoracic MPNST in the English-language literature.

Reference	Age (years)	Sex	Symptoms	Location	Metastasis	NF1	Operation	Chemotherapy	Radiotherapy (cGy)	Outcome
Elli et al., 2007 [16]	13^1^	Male	LUQ pain, weight loss	Lt paravertebral	—	Yes	Total resection	VICE, Ep, and A	Local, 4680	DOD, 14 months

Komori et al., 2003 [17]	12	Female	Chest pain, dyspnea	Rt paravertebral	N/A	No	Partial resection	N/A	N/A	DOD, 15 months

Muwakkit et al., 2006 [18]	2.5	Male	Malaise, fatigue, anorexia, and palpitations upon exertion	Rt pulmonary	N/A	No	Total resection and lobectomy	VDC	Not done	N/A

Imazu et al., 2006 [19]	12	Female	Neck swelling^2^	Rt paraclavicular	—	Yes	Total resection	Not done	Not done	NED, 12 months

Moharir et al., 2010 [20]	8	Male	Weight loss, respiratory symptoms	Lt hemithorax	—	No	Subtotal resection	Done	Not done	DOD, 2.5 months

Moharir et al., 2010 [20]	12	Female	None (incidental)	Rt paraspinal	—	Yes	Total resection	Done	Not done	NED, 2 years

Present case	16	Female	Chest pain, respiratory problem	Rt anterior	Lung	No	Total resection	VDC/IE	Rt lung and pleura, 6600	DOD, 9 months

^1^His tumor was diagnosed with MPNST associated with some angiosarcoma components.

^2^ Her tumor was located at cervicothoracic lesion.

A: actinomycin-D; C: cyclophosphamide; D: doxorubicin; DOD: died of disease; E: etoposide; Ep: epirubicin; I: ifosfamide; Lt: left; LUQ: left upper quadrant; N/A: not assessed; NED: no evidence of disease; NF1: diagnosis of neurofibromatosis type 1; Rt: right; V: vincristine.
